# Supplementary material for: Evidence supported by Mendelian randomization: impact on inflammatory factors in knee osteoarthritis
Source: Front Med (Lausanne). 2024 May 28;11:1382836. doi: 10.3389/fmed.2024.1382836 (PMC11165061; doi:10.3389/fmed.2024.1382836)
Supplement: Supplementary file 1 [file Data_Sheet_1.ZIP › data/GCST90274841_5e6/841.forest.pdf]

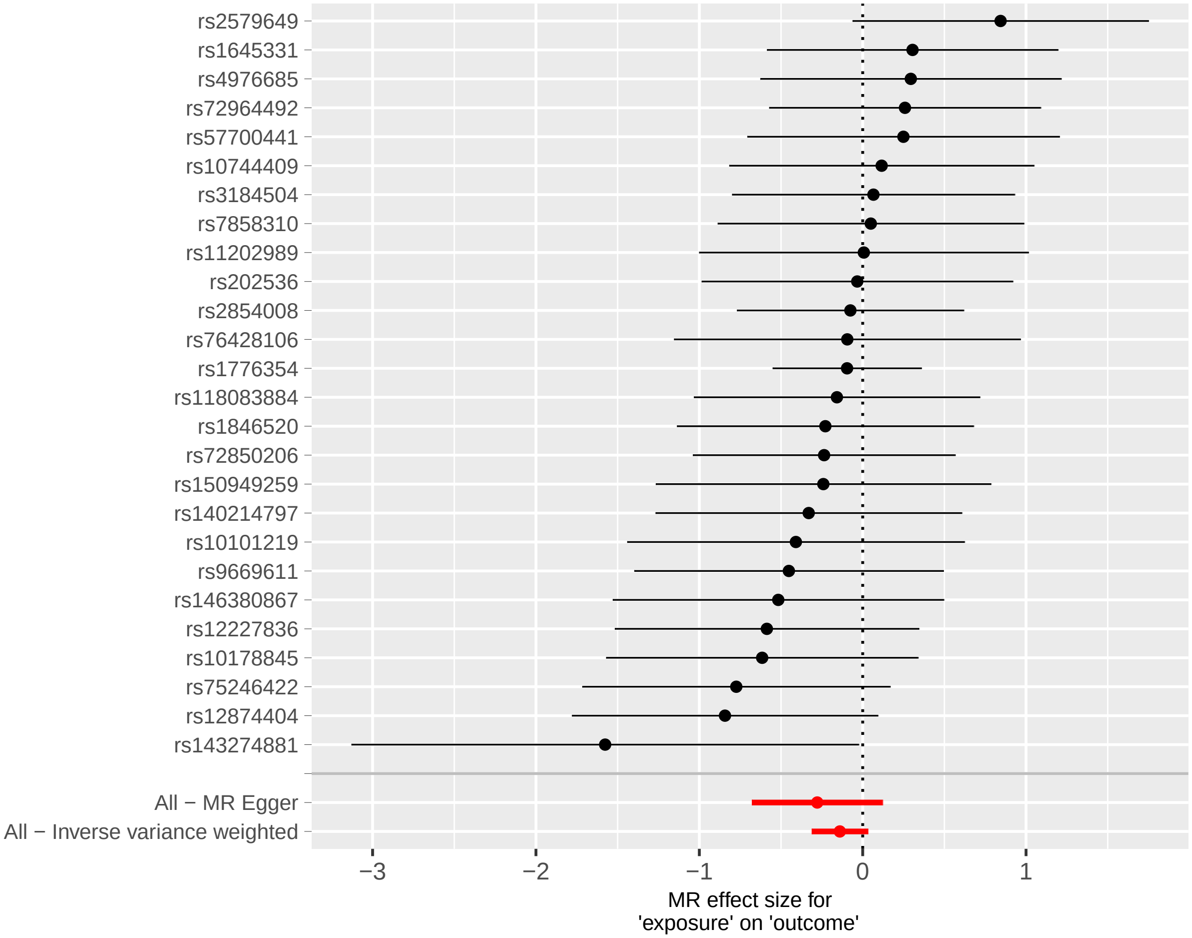
rs2579649
rs1645331
rs4976685
rs72964492
rs57700441
rs10744409
rs3184504
rs7858310
rs11202989
rs202536
rs2854008
rs76428106
rs1776354
rs118083884
rs1846520
rs72850206
rs150949259
rs140214797
rs10101219
rs9669611
rs146380867
rs12227836
rs10178845
rs75246422
rs12874404
rs143274881
All − MR Egger
All − Inverse variance weighted
−3
−2
−1
0
1
MR effect size for
'exposure' on 'outcome'
